# Conditional Inactivation of Upstream Binding Factor Reveals Its Epigenetic Functions and the Existence of a Somatic Nucleolar Precursor Body

**DOI:** 10.1371/journal.pgen.1004505

**Published:** 2014-08-14

**Authors:** Nourdine Hamdane, Victor Y. Stefanovsky, Michel G. Tremblay, Attila Németh, Eric Paquet, Frédéric Lessard, Elaine Sanij, Ross Hannan, Tom Moss

**Affiliations:** 1 Laboratory of Growth and Development, St-Patrick Research Group in Basic Oncology, Cancer Division of the Quebec University Hospital Research Centre, Edifice St Patrick, Québec, Québec, Canada; 2 Department of Molecular Biology, Medical Biochemistry and Pathology, Faculty of Medicine, Laval University, Québec, Québec, Canada; 3 Department of Biochemistry III, Biochemistry Center Regensburg, University of Regensburg, Regensburg, Germany; 4 Sir Peter MacCallum Department of Oncology, University of Melbourne, Parkville, Victoria, Australia; 5 Research Division, Peter MacCallum Cancer Centre, East Melbourne, Victoria, Australia; Indiana University, Howard Hughes Medical Institute, United States of America

## Abstract

Upstream Binding Factor (UBF) is a unique multi-HMGB-box protein first identified as a co-factor in RNA polymerase I (RPI/PolI) transcription. However, its poor DNA sequence selectivity and its ability to generate nucleosome-like nucleoprotein complexes suggest a more generalized role in chromatin structure. We previously showed that extensive depletion of UBF reduced the number of actively transcribed ribosomal RNA (rRNA) genes, but had little effect on rRNA synthesis rates or cell proliferation, leaving open the question of its requirement for RPI transcription. Using gene deletion in mouse, we now show that UBF is essential for embryo development beyond morula. Conditional deletion in cell cultures reveals that UBF is also essential for transcription of the rRNA genes and that it defines the active chromatin conformation of both gene and enhancer sequences. Loss of UBF prevents formation of the SL1/TIF1B pre-initiation complex and recruitment of the RPI-Rrn3/TIF1A complex. It is also accompanied by recruitment of H3K9me_3_, canonical histone H1 and HP1α, but not by *de novo* DNA methylation. Further, genes retain penta-acetyl H4 and H2A.Z, suggesting that even in the absence of UBF the rRNA genes can maintain a potentially active state. In contrast to canonical histone H1, binding of H1.4 is dependent on UBF, strongly suggesting that it plays a positive role in gene activity. Unexpectedly, arrest of rRNA synthesis does not suppress transcription of the 5S, tRNA or snRNA genes, nor expression of the several hundred mRNA genes implicated in ribosome biogenesis. Thus, rRNA gene activity does not coordinate global gene expression for ribosome biogenesis. Loss of UBF also unexpectedly induced the formation in cells of a large sub-nuclear structure resembling the nucleolar precursor body (NPB) of oocytes and early embryos. These somatic NPBs contain rRNA synthesis and processing factors but do not associate with the rRNA gene loci (NORs).

## Author Summary

Upstream Binding Factor (UBF) is multi-HMGB-box protein found in all vertebrates. Although this protein has been implicated in transcription of the ribosomal RNA (rRNA) gene in vitro, little is known of its function in vivo. We previously found that UBF creates a nucleosome-like structure on DNA, and that this structure is remodeled by MAP-kinase phosphorylation. Using conditional gene deletion in mouse and mouse cells we show that UBF defines the active chromatin domains of the rRNA genes and is essential for transcription of these genes. Using this system we show that, contrary to expectation, rRNA gene activity does not coordinate ribosome production. We further show that in the complete absence of rRNA synthesis a somatic nucleolar precursor body is formed. Our data show that UBF determines a dynamic transition between the active and inactive rRNA gene states that is independent of changes in DNA methylation.

## Introduction

The nucleolus is the largest visible structure in the mammalian cell nucleus and the site of ribosome biogenesis. As such, its activity is a key determinant of a cell's capacity to grow and proliferate, and its size and morphology are used as clinical markers of cancer [1]. In addition, the nucleolus is the site of assembly of ribonucleoprotein (RNP) complexes ranging from spliceosomes to telomerase, and is of key importance in mounting cellular responses to oncogenic stress [2]. The formation of the nucleolus is the result of transcription of the genes for the major ribosomal RNAs (rRNAs), the 18S, 5.8S and 28S rRNAs, which are encoded as part of the 47S precursor RNA. In mouse and human around 200 haploid copies of these genes exist as tandem repeats, the Nucleolar Organisers (NORs), at 5 chromosomal loci. Transcription of the rRNA genes is highly responsive to nutrient availability and growth factors [3] as well as the actions of oncogenes such as Myc [4] and tumour suppressors such as ARF [5]. Hence, knowledge of how the activity of these genes is determined and controlled is of fundamental importance to an understanding of cell growth, oncogenic transformation and tumour suppression.

The rRNA genes, also known as the rDNA, are transcribed by RNA polymerase I (RPI or Pol1) with the aid of the pre-initiation factors SL1/TIF1B and Rrn3/TIF1A. Recruitment of SL1 to the RPI promoter in vitro was originally shown to require Upstream Binding Factor (UBF), a multi-HMGB-box protein found in all vertebrates [3]. However, UBF is not essential for RPI transcription in vitro, and its role in the recruitment of SL1 has more recently been questioned [6], [7]. Further, UBF displays almost no DNA sequence selectivity [8]–[10] and is found widely dispersed throughout the rDNA repeat, suggesting that, rather than functioning as a pre-initiation factor, it may play an epigenetic role in the formation and maintenance of active rRNA gene chromatin [11], [12]. Consistent with this, UBF binding is maintained during metaphase only at NORs that were active in the previous cell cycle, and this binding predicts their continued transcriptional activity in subsequent cell cycles [13]–[16]. In vitro, UBF binds DNA as a dimer and uses its HMGB-boxes to induce six in-phase bends, generating a single 360 deg. loop of DNA of about 140 bp in length, a structure we refer to as the rDNA Enhancesome [8], [17], [18]. The Enhancesome resembles the histone nucleosome in both its size and protein-DNA composition, but the two structures are fundamentally different and could not co-exist at the same site. On the other hand, UBF can bind to core nucleosomes in vitro without disrupting them [19]. This said, enhanced recruitment of UBF to the endogenous human rRNA genes correlates with a proportionate reduction in core histone binding at the same sequences, suggesting that in vivo UBF predominantly replaces nucleosomes [20]. Further, Hmo1, the ortholog of UBF [21], was shown to fully replace the core histones on active yeast rRNA genes [3], [22].

MAP-kinase phosphorylation of the UBF was found to regulate RPI transcription elongation rates in vitro and in vivo in the response to growth factors [23]–[26]. Despite this, the requirement for UBF in rRNA gene activity is still uncertain, and partial depletion of mouse UBF did not significantly affect rRNA synthesis rates [12]. Further, the yeast UBF ortholog Hmo1 is encoded on a non-essential gene [21], [27], suggesting that it plays an assisting rather than a key role in RPI transcription. To definitively determine the in vivo requirements for UBF, we have studied conditional inactivation of the mouse *Ubf* gene, and in so doing have made several unexpected findings. We find that the mouse *Ubf* gene is indeed essential for rRNA gene activity, cell proliferation and embryo development. Elimination of UBF causes large-scale changes in rRNA gene chromatin consistent with a transition from the active state to a potentially active resting state, but not heterochromatinization. Unexpectedly, inactivation of rRNA gene activity has no effect on the activity of the hundreds of RPII and RPIII genes implicated in ribosome biogenesis, showing that rRNA gene activity does not coordinate the gene expression required for ribosome assembly. Finally, elimination of UBF reveals somatic nucleolar precursor bodies that are spatially distinct from chromosomal rDNA loci.

## Results

### UBF is essential for mouse development

**Figure 1:**
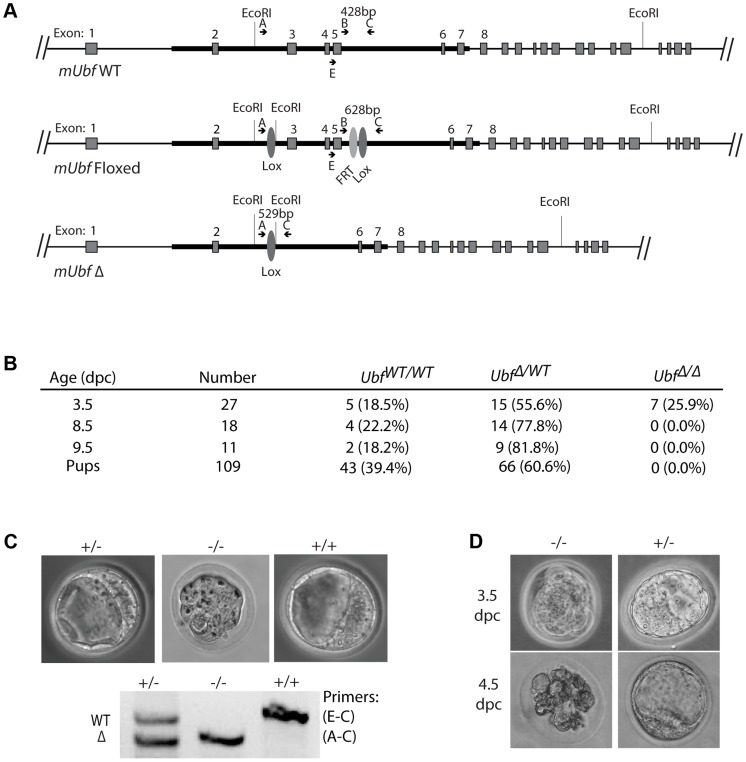
The *Ubf* gene is essential for mouse development beyond morula. A) Structure of the wild type *Ubf* (WT), the conditional *Ubf^−fl^* (Floxed), and the deleted *Ubf* ^−Δ^ (Δ) alleles. B) Survival statistics for *Ubf^wt/wt^*, *Ubf* ^Δ*/wt*^ and *Ubf* ^Δ/Δ^ mouse embryos and offspring. Note that no embryos were detected at or after 8.5 dpc. C) Examples of mouse embryos and genotyping at the equivalent of 3.5 dpc. *Ubf* null embryos arrest at the morula stage. D) In vitro development of 3.5 dpc embryos to late blastocysts (equivalent to 4.5 dpc). *Ubf* null embryos do not develop further and necrose.

To establish the in vivo requirements for UBF, we generated mouse embryonic stem (ES) cells carrying a potentially conditional “flox-neo” *Ubf* allele in which Lox recombination sites were placed in intron 2 and intron 5, and a *neo* selective marker gene flanked by FRT sites was inserted within intron 5 (Figure S1A & B). Mice from two independent ES cell lines heterozygous for the *Ubf^fl-neo^* allele were used to generate two mouse lines that subsequently displayed indistinguishable phenotypes. Mice heterozygous for the *Ubf^fl-neo^* allele were viable, but no mice homozygous for this allele were identified (data not shown), suggesting that the *Ubf* gene was inactivated by the insertion and hence was essential. The mice were then crossed with FLPeR (Flipper) and Cre expressing mice to generate both *Ubf^fl^* and *Ubf* ^Δ^ alleles, and subsequently Cre and Flipper transgenes removed by backcrossing (Figure 1A and S1B). While *Ubf^fl/fl^* mice appeared normal and were fully viable, no *Ubf-null* pups were identified (Figure 1B). Analysis of embryos at prenatal 9.5 dpc and 8.5 dpc also failed to detect *Ubf* ^Δ/Δ^ embryos, though *Ubf* ^Δ*/wt*^ heterozygotes were detected at a near Mendelian ratio. At 3.5 dpc *Ubf* ^Δ/Δ^ embryos were detected, but were systematically arrested at morula (∼2.5 dpc), at or during the compaction phase (Figure 1C and S1E). By contrast, *Ubf* ^Δ*/wt*^ and *Ubf^wt/wt^* litter-mates displayed a normal trophectoderm (TE) layer, inner cell mass (ICM) and blastocoel cavity. When the *Ubf* ^Δ/Δ^ embryos were cultured *in vitro* they failed to develop further, while *Ubf* ^Δ/*wt*^ and *Ubf^wt/wt^* litter-mates developed to form late blastocysts (Figure 1D and S1F). Thus, the UBF gene is required for embryo development beyond the morula stage, that is, very soon after the normal onset of rRNA gene transcription [28].

### UBF is essential for transcription of the rRNA genes in vivo

**Figure 2:**
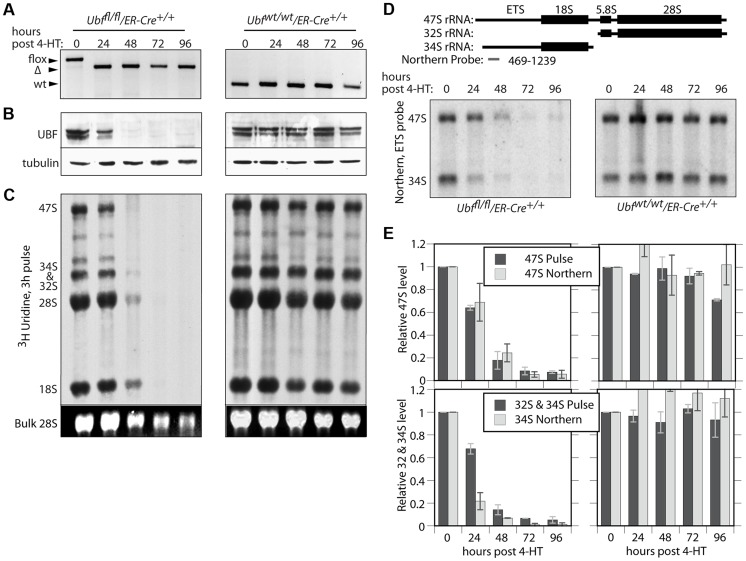
UBF is essential for the synthesis of the major rRNAs in cell culture. A) to E) *Ubf^fl/fl^/Er-cre^+/+^* and *Ubf^wt/wt^ Er-cre^+/+^* cells were treated with 4-HT to induce recombination in the *Ubf* gene and at the indicated time points, A) genotyped for *Ubf* recombination, B) analyzed by Western blot for UBF levels, and C) metabolically labelled with [^3^H]-uridine to follow rRNA synthesis of the rRNAs and their precursors. D) Northern blot analysis of the 47S and 34S rRNA pools. The upper diagram shows the organisation of the larger rRNA precursors and the probe used. In C) “Bulk” refers to the EtBr stained total RNA fractionation. E) Quantitative analyses of rRNA synthesis rates and pool sizes.

To determine whether or not UBF was required for transcription of the rRNA genes, we derived cell lines conditional for UBF expression from *Ubf^fl/fl^* mice (Figure 1A) carrying a Tamoxifen (4-HT) inducible ER-Cre recombinase [29]. Mouse embryonic fibroblasts (MEFs) were then isolated from homozygous *Ubf^fl/fl^/Er-cre^+/+^* mice and from isogenic *Ubf^wt/wt^/Er-cre^+/+^* control mice and immortalized by transfection with an SV40-Tt expression vectors (iMEFs). Short-term, induction of ER-Cre activity by a 4 h treatment with 50 nM 4-HT induced near complete excision of the floxed UBF exons by 12 h, and excision was complete by 24 h post 4-HT (pHT) (Figure 2A and S2A). Though UBF protein levels were already significantly reduced by 24 h pHT, metabolic pulse labeling and Northern blot both revealed only a small effect on rRNA synthesis (Figure 2B to E), as observed for siRNA knockdown [12]. However, by 48 h pHT UBF protein was practically eliminated and this corresponded to near complete arrest of rRNA synthesis, and by 72 h pHT synthesis was no longer detected. UBF elimination had no significant effect on the levels of other proteins believed to be essential for rRNA synthesis or processing (RPI(A194), Rrn3/TIF1A, TBP, TAF1B & -C, TTF1 & fibrillarin), or on processing of the 47S rRNA (to be discussed later) and cells also did not display signs of stress such as enhanced p53 or MDM-2 levels (Figure S2B and C). Inhibition of rRNA synthesis was therefore the direct result of the elimination of UBF. It is important to underline that this is the first demonstration of a strict requirement for UBF in the transcription of the rRNA genes.

### UBF associates specifically with the rRNA gene enhancer and 47S transcribed regions

**Figure 3:**
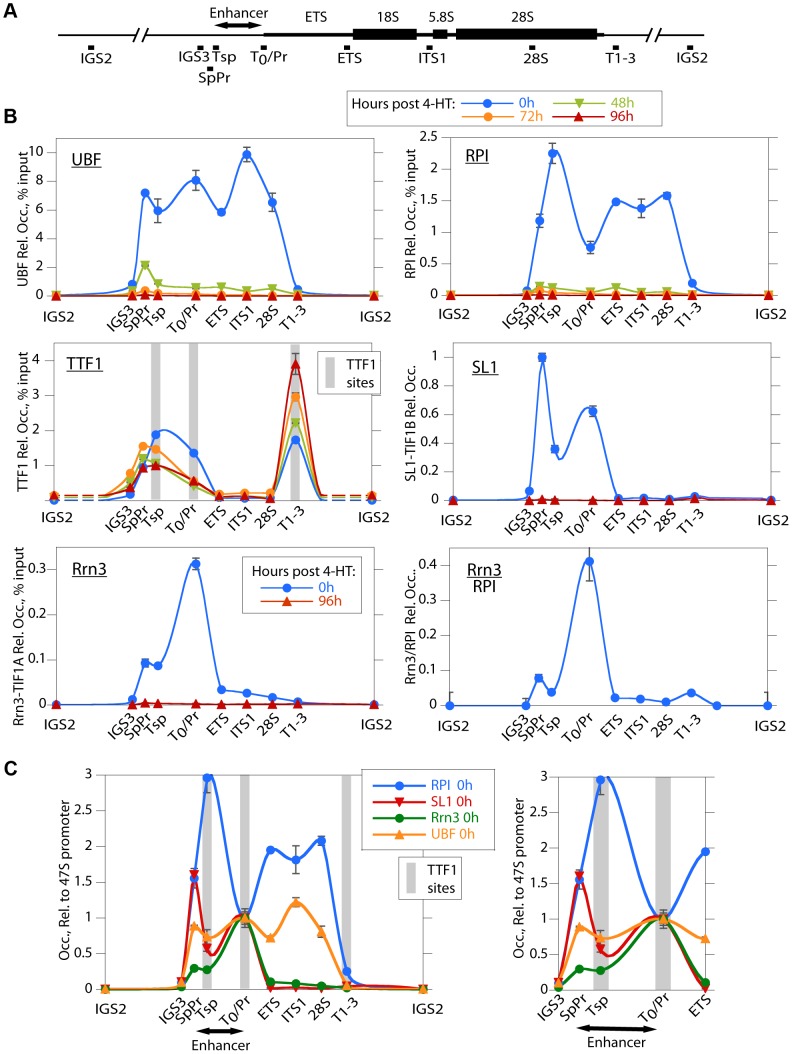
UBF elimination causes release of the RPI and all RPI initiation factors, but not of the termination factor TTF1. A) Map of the rDNA repeat showing above; the coding and Enhancer regions, and below; the amplicons sampled by ChIP analysis. B) Association of UBF, RPI, TTF1, SL1 (TIF1B) and Rrn3 (TIF1A) and of initiation competent RPI (Rrn3/RPI) determined by ChIP/Q-PCR assays of chromatin from *Ubf^fl/fl^/Er-cre^+/+^* cells prepared at the indicated times pHT. C) Relative distribution of RPI, SL1, Rrn3 and UBF at 0 h pHT, i.e. before *Ubf* recombination. Left panel shows data on an enlarged horizontal scale. In B and C the horizontal axes indicate the position of amplicons, and the grey bars the positions of known binding sites for TTF1. *Ubf* gene recombination and UBF protein levels were assayed in parallel with ChIP analyses and followed those shown in Figure 2A and B. The data shown in B and C are given after subtraction of the parallel preimmune Control ChIP data. They are derived from single ChIP preparations analyzed in triplicate, but are representative of the data from biological replicates, see Materials and Methods.

To investigate the reasons for the arrest of rRNA synthesis on UBF loss, we analyzed the association of RPI and RPI pre-initiation factors with the rRNA genes (the rDNA). As expected, in the untreated *Ubf^fl/fl^/Er-cre^+/+^* cells, UBF associated with the rDNA at the 47S promoter (T0/Pr), throughout the transcribed region and at the Spacer Promoter (SpPr) and Spacer Terminator (Tsp) lying ∼2 kbp upstream of the 47S promoter, but not across the rest of the Intergenic Spacer (IGS) (Figure 3A and B). By 48 h pHT, only a residual level of UBF remained associated with the rDNA and by 72 and 96 h pHT UBF association was undetectable. This confirmed that UBF association is limited to the transcribed and enhancer regions of the mouse rDNA and therefore parallels the localization of Hmo1 within the yeast rDNA [30].

### UBF is necessary for the formation of the RPI initiation complex and RPI recruitment

Consistent with the near complete shutdown of rRNA synthesis (Figure 2C), by 48 h pHT association of RPI with the rDNA was hardly detected, and by 72 h pHT it was eliminated (Figure 3B). Thus, RPI and UBF levels on the rDNA reduced in step with each other. Rrn3/TIF1A, an essential factor associated with the initiation competent RPI, colocalized with RPI at the 47S promoter in untreated cells and was also completely eliminated along with UBF, as were the subunits of the SL1 pre-initiation complex, (TBP, TAF1B/TAFI68 and TAF1C/TAFI95), (Figure 3B and S3B). Thus, UBF was not only essential for recruitment of initiation competent RPI, but also for the formation and/or maintenance of the pre-initiation complex. Our data, therefore support the notion that UBF is required for the recruitment of SL1 to the 47S promoter in vivo, and underline UBF's fundamental importance in determining rRNA gene activity.

### Association of the transcription termination TTF1 with the rDNA is independent of UBF

In contrast to the other RPI transcription factors, association of the Transcription Termination Factor (TTF1) with the rDNA was clearly not dependent on UBF (Figure 3B). However, elimination of UBF did cause a decrease in TTF1 association with the Spacer Promoter (SpPr), Spacer Terminator (Tsp) and 47S promoter proximal terminator (T_0_). In contrast, its association with the 47S termination sites (T1–3) was strongly enhanced. This suggests that TTF1 is predominantly a constitutive factor, found on both active and inactive genes. Its partial displacement from the binding sites upstream of the 47S transcribed region to the downstream termination sites may be related to its function in looping the rDNA [31], but other scenarios are possible.

### Recruitment of RPI to the Enhancer (Spacer) Promoter and 47S promoter are distinctly different

The rDNA sequences between the Spacer Promoter and the 47S promoter act *in cis* as enhancers of gene transcription [32]–[36]. However, the mechanism underlying their action is still not understood. We noted that UBF elimination abrogated binding of RPI, Rrn3/TIF1A and SL1 to the Spacer Promoter, as it did to the 47S promoter. However, comparison of the binding profiles of RPI and Rrn3/TIF1A at the Spacer and 47S promoters showed distinct differences. RPI was 5 times less likely to be associated with Rrn3 at the Spacer Promoter than at the 47S promoter (see Rrn3/RPI in Figure 3B). Further, the major peak of RPI within the spacer mapped to the Spacer Terminator (Tsp) and not to the Spacer Promoter (SpPr). In contrast, SL1 preferentially mapped to the SpPr and its ChIP signal was low at the Tsp, this can be seen more clearly in Figure 3C. Since, after initiating transcription RPI releases Rrn3, the data suggest that most RPI in the spacer is engaged in transcription but arrested at the Spacer Terminator. In fact, more RPI maps to the Tsp than to either the SpPr or the 47S promoter, suggesting that a majority of genes have RPI arrested at the upstream edge of the enhancer repeats. Why this should be so is unclear, but is possibly related to DNA looping between Tsp and T_0_ or the formation of an upstream barrier to the spread of repressive chromatin.

### Loss of UBF induces changes in the rDNA chromatin, but not CpG methylation

**Figure 4:**
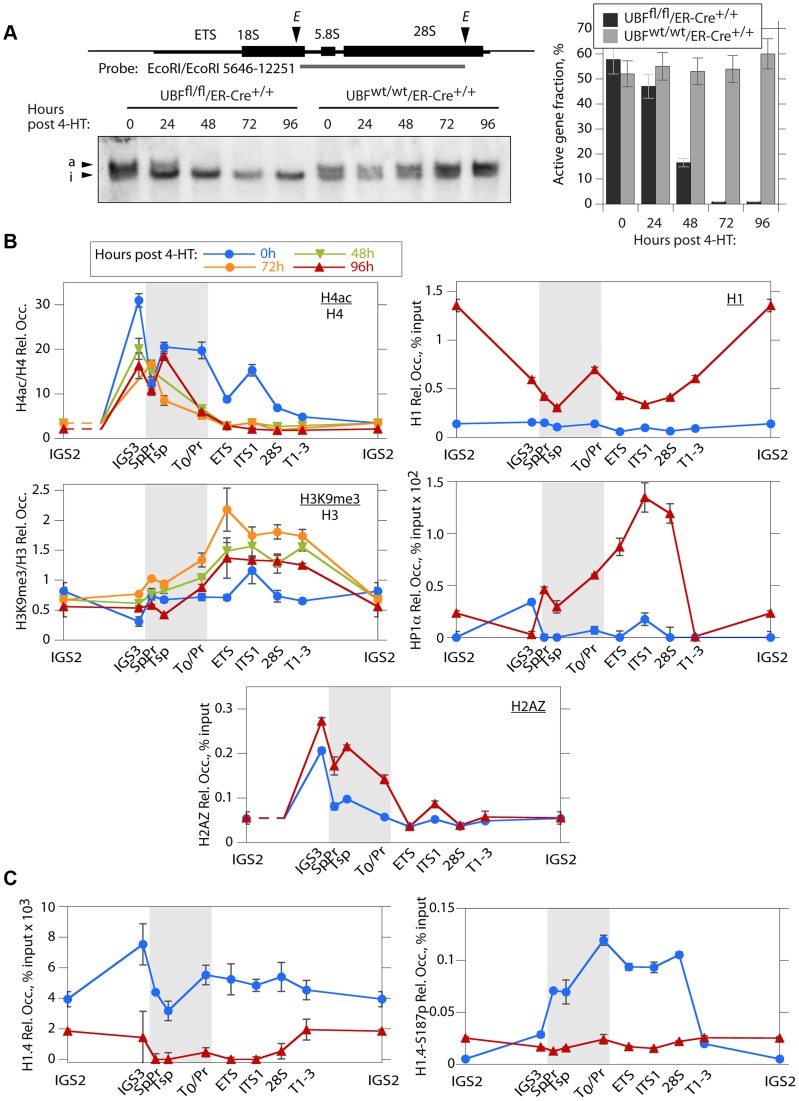
The rDNA chromatin is extensively remodelled during UBF elimination. A) Psoralen crosslinking analysis of the rRNA genes in *Ubf^fl/fl^/Er-cre^+/+^* and *Ubf^wt/wt^/Er-cre^+/+^* cells at the indicated times pHT. The probe position is indicated in the upper diagram, and the lower panel shows a typical electrophoretic separation of actively transcribed “a” and inactive “i” genes. The right-hand panel displays a quantitation of the fraction of transcriptionally active and inactive genes at each time point. Elimination of UBF at 48 h pHT correlates with full inactivation of the rRNA genes. B) and C) Changes in histone modifications penta-acetyl H4 (H4ac) and H3K9me3, and relative Histone H1, H2AZ, H1.4, H1.4-S187p and chromosomal protein HP1α levels associated with the rDNA during UBF elimination (hours pHT). The grey band indicates the extent of the rRNA gene Enhancer sequences. Mapping of amplicons is as in Figure 3A. Again here *Ubf* gene recombination and UBF protein levels were assayed in parallel and closely followed those shown in Figure 2A and B. The data shown in B and C are given after subtraction of the parallel preimmune Control ChIP data. They are derived from single ChIP preparations analyzed in triplicate, but are representative of the data from biological replicates, see Materials and Methods.

The mouse genome contains about 200 rRNA genes, only a fraction of which are actively transcribed. This active fraction is characterized by its enhanced accessibility to psoralen crosslinking [37], [38]. As expected, the active gene fraction was found to parallel the level of UBF and RPI association, and by 48 h pHT very few genes remained active (Figure 4A). UBF elimination also corresponded with an abrupt reduction in the association of the active chromatin marker H4ac5, (H4ac) and an increase in the repressed chromatin marker H3K9me3, particularly over the transcribed region of the rDNA, changes that were not detected at the actively RPII-transcribed gene Camk2b (Figure 4B and S4B & D). UBF elimination further induced a significant increase in the recruitment of the linker histone H1 throughout the rDNA repeat, but especially at the 47S promoter (T_0_/Pr) and within the IGS. Association of the heterochromatic HP1α with the rDNA was also enhanced but unlike H1 this occurred over the enhancer and transcribed regions previously occupied by UBF (Figure 4B and S4C). However, no corresponding change in the CpG methylation status of the rDNA was detected (Figure S4A), suggesting that UBF elimination did not induce a more permanent heterochromatinization. Consistent with this, the level of H4ac remained high over the spacer promoter, spacer terminator and the immediately adjacent IGS sequences (IGS3), (see again Figure 4B and S4B), and corresponded with the enhanced recruitment of H2A.Z and the maintenance of TTF1 binding to these regions (Figures 3B and 4B). H2A.Z is known to mark promoters of potentially active genes [39]–[42]. Thus, the lack of enhanced DNA methylation and the maintenance of TTF1, H2A.Z and H4ac over the rRNA gene enhancer indicates that the rRNA genes can remain in a poised state even in the absence of UBF. This suggests that one function of the enhancer region is to define or maintain a pool of potentially active genes.

### H1 variant H1.4 associates with the active rDNA in a UBF-dependent manner

H1.4-S187p, a phosphorylated form of the H1 variant H1.4, was previously shown to be enriched on the human rDNA during interphase, suggesting that unlike the canonical H1 it may be permissive to rRNA transcription [43]. We found that H1.4 also bound throughout the mouse rDNA, and contrary to canonical H1, this binding was strongly suppressed by UBF elimination (Figure 4C). Further, phospho-H1.4 (H1.4-S187p) mapped specifically to the enhancer and transcribed regions and was lost on UBF elimination (Figure 4C and S4C). Thus, H1.4 and especially H1.4-S187p selectively bind to the transcriptionally active rRNA genes.

### UBF is not required for 47S rRNA processing nor for 5S rRNA or U3 RNA synthesis

Ribosome biogenesis is a highly coordinated process that depends on the expression of many hundreds of genes and on a complex series of assembly and processing events [44]. The expression of the ribosomal proteins (r-proteins) is coordinately regulated with rRNA synthesis in yeast [45], and the rate of synthesis of the pre-rRNA is the determinant regulatory factor in the expression of r-proteins in E. coli [46], [47]. We, therefore, expected that shutdown of *de novo* rRNA synthesis following UBF elimination would affect the expression of a broad range of genes. The first indication that this might not be so came from the observation that processing of residual 47S rRNA appeared to continue normally as determined by 32/34S levels in metabolic labeling and Northern analysis (Figure 2C–E). In particular, at 48 h pHT, when UBF was essentially absent, the residual pre-rRNA still being synthesized was processed into the 18S and 28S and 32/34S in the same proportions as at 0 h pHT (Figure 2C & E). This argued that UBF might not play such a significant role in rRNA processing as previously suggested [48], [49]. More importantly, it suggested that transcription of the major rRNA genes might not in fact be implicated in regulating other products needed for ribosome biogenesis.

**Figure 5:**
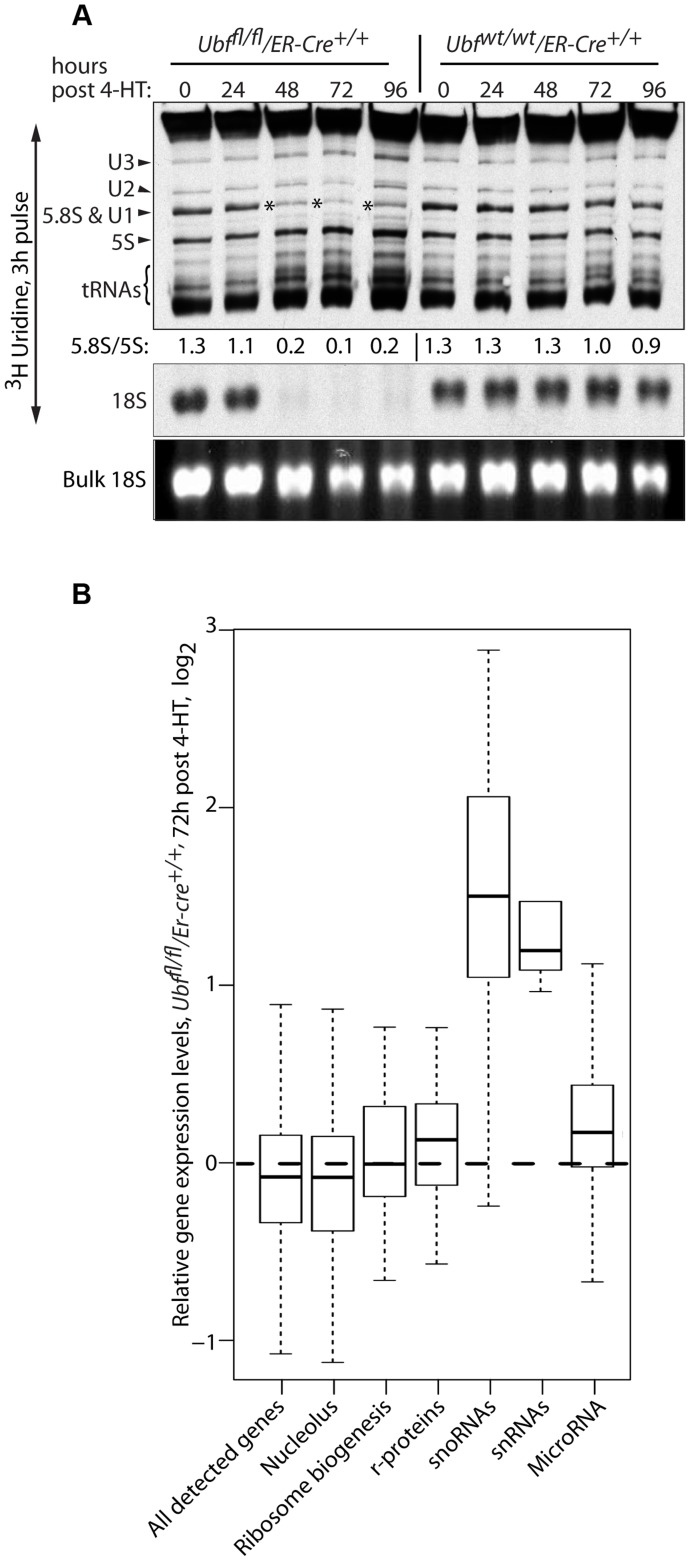
UBF elimination has only minor effects on global gene expression. A) Metabolic labeling of *Ubf^fl/fl^/Er-cre^+/+^* and *Ubf^wt/wt^/Er-cre^+/+^* cells with [^3^H]-uridine to follow synthesis of U2 and U3 snRNAs, 5.8S rRNA, 5S rRNA, tRNAs and 18S at the given time points pHT. “Bulk 18S” refers to the EtBr input control. The ratio of incorporation into 5.8S relative to 5S (5.8S/5S) is given below each track of the upper panel. B) Boxplot of unbiased expression microarray data for various gene classes. The plots indicate the relative gene expression levels for *Ubf^fl/fl^/Er-cre^+/+^* cells at 72 h pHT relative to levels for *Ubf^wt/wt^/Er-cre^+/+^* at the same time point. The original data can be found in the GEO databank under the accession number GSE55450.

The 5S rRNA is an essential component of the ribosome, and hence its expression would be expected to be coordinated with that of the major rRNAs. However, neither 5S rRNA nor indeed tRNA synthesis was suppressed by the shut-down of major rRNA synthesis (Figure 5A). Metabolic labeling showed that both 5S rRNA and tRNA synthesis was maintained and indeed enhanced for at least 48 h after the complete shutdown of 18S (and 28S) production. By comparison, synthesis of 5.8S rRNA, which is processed from the 47S pre-rRNA, followed that of the other major rRNAs. Consistent with continued pre-rRNA processing in the absence of UBF, metabolic labeling also showed that the U3 processome RNA continued to be synthesized, as did the U1 and U2 splicing RNAs. Thus, quite unexpectedly synthesis of these small RNAs by RPII and RPIII was not affected by UBF elimination, nor by the shut-down of major rRNA synthesis.

### UBF elimination does not significantly affect expression of ribosome biogenesis genes

Unbiased expression-microarray analysis of *Ubf^fl/fl^/Er-cre^+/+^* and control *Ubf^wt/wt^/Er-cre^+/+^* cells at 0, 48, 72 and 96 h pHT showed that UBF elimination caused no significant changes in mRNA levels, e.g. see data for 72 h pHT (Figure 5B and S5). In particular, we detected no significant changes in r-protein gene expression, or in the expression of the majority of other genes implicated in ribosome biogenesis. Even the genes encoding components of the RPI transcription machinery (RPI and SL1 subunits, Rrn3/TIF1A and TTF1) displayed no significant change in expression, the sole exception being TAF1D (TAFI41) [50], whose mRNA was enhanced about 3 fold at 72 h pHT. In contrast, the levels of the snoRNAs, responsible for directing rRNA modifications, increased very significantly, as did the snRNAs including the U3 processome RNA and the U1 and U2 splicing RNAs, in agreement with the metabolic labeling data (Figure 5A). To a lesser extent some miRNAs including the X-linked Mir-18, -19 [51] and Mir-337 [52] also showed a small increase. Together with the findings of continued rRNA processing and the observation of active synthesis of 5S, tRNA and other small RNAs by both RPII and RPIII, these data argue strongly against a role for 47S rRNA synthesis in coordinating the expression of genes implicated in ribosome biogenesis.

### Nucleolar factors congregate in large nucleolar bodies after UBF elimination

**Figure 6:**
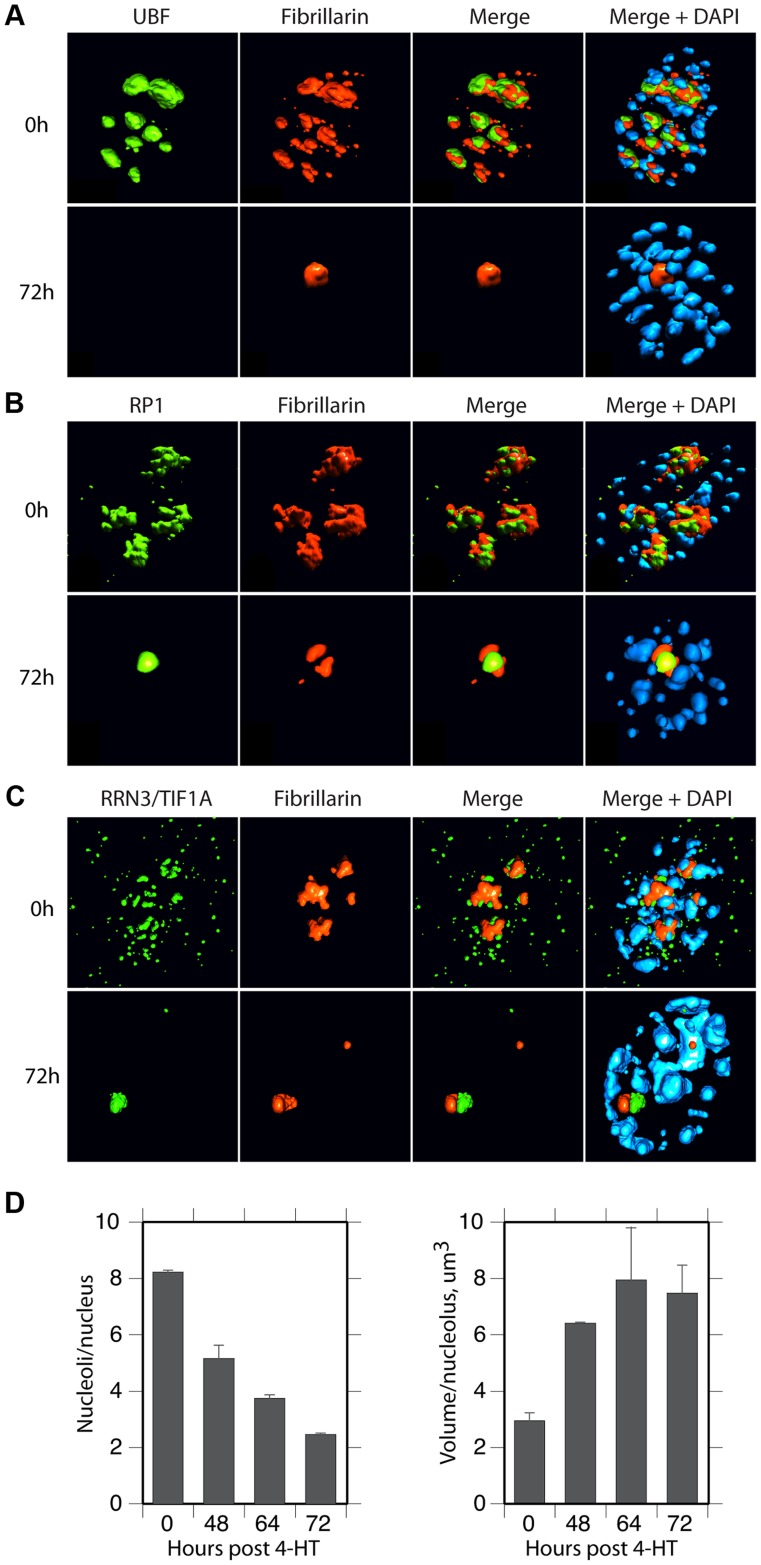
UBF elimination reveals that nucleolar protein bodies exist independently of rRNA gene activity. A) to C) 3D IF analysis of respectively UBF, RPI (large subunit, A194) and Rrn3/TIF1A relative to Fibrillarin and DNA stained with DAPI. The subnuclear volumes of the indicated proteins are indicated as surfaces of constant fluorescence intensity (isosurface) with directional pseudo-illumination to indicate their 3D form. D) Left; quantitation of the mean number of (Fibrillarin positive) nucleoli or nucleolar bodies per nucleus, and right; mean (Fibrillarin) volume of individual nucleoli. Data were the mean of three independent series of IF analyses, and ∼20 nuclei were analyzed per time point.

**Figure 7:**
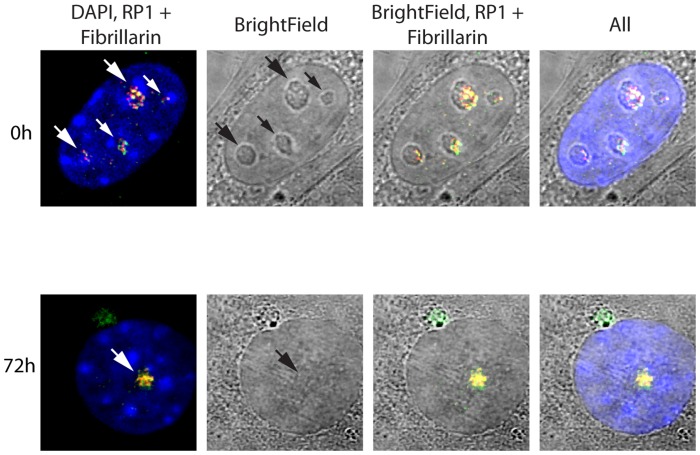
Somatic Nucleolar Precursor Bodies are distinguished from nucleoli by not being visible in bright field. IF and bright field images of *Ubf^fl/fl^/Er-cre^+/+^* cells at 0 and 72 h pHT counterstained with DAPI and showing the location of RPI (red) and fibrillarin (green).

The spatial localization of the nucleolar factors RPI, Rrn3/TIF1A, TTF and Fibrillarin was followed in *Ubf^fl/fl^/Er-cre^+/+^*cells at various time points after 4-HT treatment. All these factors coalesced into dense nucleolar bodies (Figure 6A–C and S6A–D). This nucleolar reorganization was complete by 72 h pHT, just 24 h after complete arrest of rDNA transcription, but was not observed in 4-HT treated *Ubf^wt/wt^/Er-cre^+/+^* control cells. Analysis of 3D image stacks showed that the number of distinct nucleoli or nucleolar bodies reduced in step with UBF depletion, such that by 72 h pHT, when UBF was no longer detected, an average of two nucleolar bodies per nucleus remained (Figure 6D). Each of these nucleolar bodies was significantly larger and more intensely labeled than the original nucleoli (Figure 6D), suggesting that they were each formed by the coalescence of several nucleoli. However, interphase nucleoli are highly visible as large sub-nuclear structures even in bright-field contrast images, while these nucleolar bodies were not visible in bright-field (Figure 7), suggesting that, in agreement with the complete depletion of rRNA processing intermediates, they no longer contained pre-ribosomal particles (Figure 2D). A similar situation pertains during mitosis, when the “bright-field” nucleolus appears to disperse, and in mammalian oocytes and early embryos in which the extremely large nucleolar precursor body is not evident in bright-field, e.g. [28], [53].

### Nucleolar bodies are not associated with the rRNA gene loci

**Figure 8:**
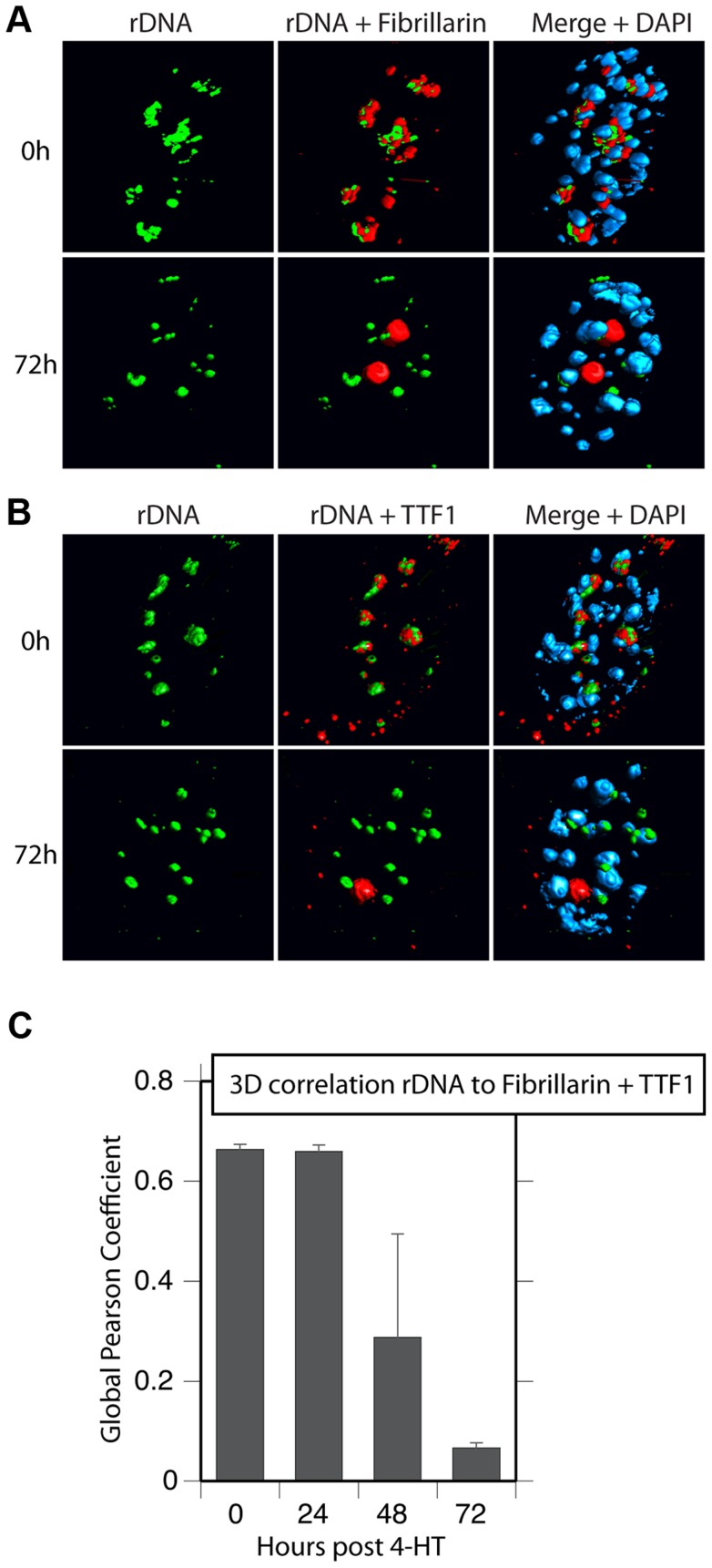
Nucleolar bodies are spatially distinct from the rDNA and NORs. A) and B) 3D Immuno-FISH analysis of the spatial distribution of rDNA relative to Fibrillarin and TTF1 at the indicated times pHT treatment. The subnuclear volumes of the indicated proteins and DNA are indicated as surfaces of constant fluorescence intensity (isosurface) with directional pseudo-illumination to indicate their 3D form. C) Quantitative analysis of the 3D spatial correlation of rDNA FISH fluorescence with Fibrillarin and with TTF1 fluorescence. Data were the mean of two independent Immuno-FISH analyses in which >20 nuclei were analyzed.

Surprisingly, 3D immuno-FISH revealed that the nucleolar bodies forming on UBF elimination did not in fact contain the rRNA genes at all. Rather the rRNA genes existed in small, discrete nuclear foci (Figure 8A & B and S7B to D) whose number suggested that they represented individual NORs (e.g. compare 72 h images in Figure 8A & B with the mitotic spread in Figure S7A). Quantitative analysis of the rRNA gene FISH signal and the TTF1 and Fibrillarin immunofluorescence in 3D image stacks (Figure 8C) confirmed that by 72 h pHT there was essentially no co-localization of the rRNA genes with the nucleolar bodies. The separation of rRNA genes from the nucleolar bodies also agreed with the complete loss of RPI and TIF1A interaction with the rDNA after UBF elimination, as shown by ChIP (Figure 3). Though TTF1 was both in the nucleolar bodies (Figures 8, S6 and S7) and on the rRNA genes (Figure 3B) at 72 h pHT, it is the only RPI factor that targets specific DNA sequences with high affinity [31], and is in large excess over its rDNA binding sites [5]. To our knowledge, these data are the first to identify the existence in somatic cells of large sub-nuclear structures able to sequester nucleolar proteins independently of rDNA loci or the rRNA genes.

## Discussion

UBF has been termed an “architectural” protein due to its unique ability to induce a complex similar in size and protein-DNA content to the nucleosome of histone chromatin [54]. The discovery of this structure, its modulation by ERK phosphorylation, and the finding that UBF is broadly distributed across the rDNA repeat [8], [11], [24], [25], have provided a potential explanation for growth factor regulation of RPI elongation rates in vivo [3]. However, knockdown of UBF by some 80% failed to significantly affect rRNA synthesis [12] and its role in vitro as a RPI transcription factor has also been questioned. Using conditional deletion mutation in MEFs we have now demonstrated that UBF is indeed essential for rRNA gene transcription, as well as for the formation of the RPI pre-initiation complex and the active epigenetic state of these genes. Our data further show that nucleolar proteins congregate in large Nucleolar Precursor Bodies that are spatially distinct from rDNA loci.

We found that homozygous deletion of the *Ubf* gene in mouse embryos arrested development at morula. This is soon after the onset of rDNA transcription and suggests that the maternal ribosome pool is limiting for development beyond this stage. Consistent with this, embryos carrying homozygous deletions of the genes encoding the second largest subunit of RPI [55] and the processome component Fibrillarin [56] also arrest development at this stage. In stark contrast, homozygous deletion of the gene for RPI initiation factor Rrn3/TIF1A was found to arrest development at 7.5–9.5 dpc [57], by which stage the ribosome content of the embryo is many thousands of times the maternal component. It is unclear why this should be so and we are presently trying to understand this discrepancy.

Elimination of UBF in cell culture caused the complete arrest of RPI transcription and disruption of the RPI preinitiation complex. It also induced partial heterochromatinization of the rDNA, as indicated by enhanced K9 tri-methylation of histone H3, and a degree of recruitment of HP1α and of canonical histone H1. However, certain markers of potential gene activity were maintained. Penta-acetyl H4 levels remained significant over the RPI promoter and the upstream Enhancer and the H2A.Z level over these regions was enhanced. Consistent with this, no increase in CpG methylation of the rDNA was detected despite continued binding of the chromatin remodeller TTF1, known to recruit remodeling complexes containing DNA methyltransferases. UBF was previously shown to displace H1 from nucleosomes in vitro [19]. We were, therefore, surprised to find that binding the phosphorylated form of the H1 variant H1.4 (H1.4-S187p) was dependent on UBF and colocalized with it and with the polymerase. In the light of this and the previous data from the Mizzen group showing the correlation of H1.4-S187p with rRNA transcription in human [43], the classification of H1 variants as heterochromatin proteins clearly requires some re-evaluation.

**Figure 9:**
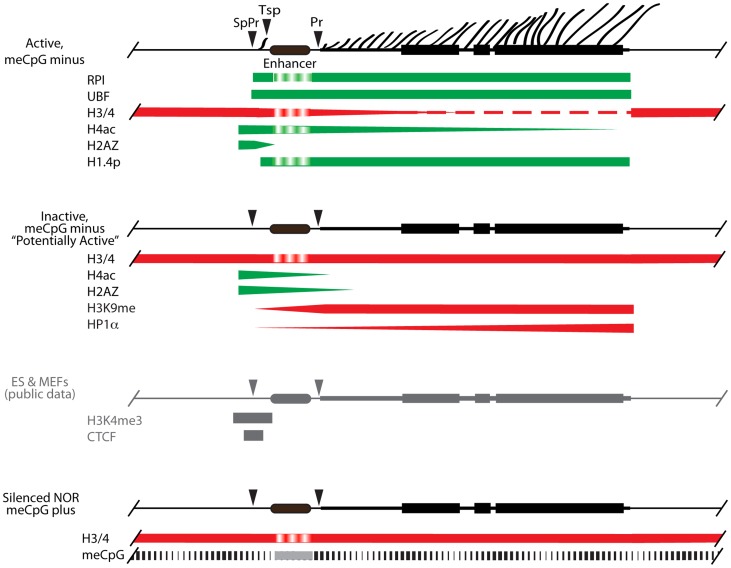
A model for the chromatin structure of the three distinct states of rRNA gene activity. Active, unmethylated (meCpG minus) genes maintain UBF and phospho-H1.4 (H1.4p) over their Enhancer region and their 47S transcribed gene region (indicated by lateral fibrils and rRNA gene blocks), while RPI is restricted to the 47S region and to a site close to the spacer terminator (Tsp), the latter probably in an arrested elongation state. These same gene regions display variable levels of H4ac, and are denuded of nucleosomes. H2Az is most likely present adjacent to the spacer promoter (SpPr), while the preinitiation complex SL1/TIF1B is present at both spacer and 47S Promoters (Pr), (not shown). The Inactive, unmethylated (meCpG minus) fraction of genes is devoid of UBF, displays enhanced occupation by H3K9me3 and HP1α, especially towards the 3′ 47S region, and nucleosomes replace UBF over most of its interaction domain. But these inactive genes retain H2Az and H4ac over the SpPr and Enhancer regions, suggesting they are in dynamic exchange with the actively transcribed gene population. Our unpublished alignments of H3K4me3 and CTCF Encode data sets for ES and MEFs show that these factors are also present flanking the SpPr probably on both Active and Inactive unmethylated genes. The methylated (meCpG) gene fraction is unaffected by UBF loss, and most probably exists in a classical heterochromatic state corresponding to the inherited constitutively silenced NORs. Due to limited data, the status of the “Enhancer” region is difficult to ascertain and this is indicated by a different shading.

The ∼200 haploid rRNA genes exist in one of three states dependent on their activity and methylation status [20], [58]. Our previous data suggest that over 50% of genes display no DNA (CpG) methylation, and these unmethylated genes exist in two states, either actively transcribed or silent. A third group of genes is heavily CpG methylated and probably correspond to the constitutively silenced NORs. Since UBF elimination does not enhance CpG methylation, but shuts down all transcription, the ChIP data (Figures 3 & 4) provide a window on the chromatin status on the unmethylated silent genes and suggest that they exist in a potentially active state (Figure 9). Active genes are engaged by UBF and H1.4-S187p throughout the enhancer and 47S coding regions, the preinitiation complex SL1/TIF1B is bound at both 47S (Pr) and Spacer (SpPr) Promoters, RPI is engaged in ternary (elongating) complexes throughout the 47S region, and a ternary RPI complex is arrested at or near the Spacer Terminator (Tsp). The unmethylated inactive genes have no UBF, H1.4S-187p, SL1 or RPI, but retain H4ac5 and H2AZ over the SpPr/Enhancer region, and accumulate both H3K9me3 and HP1α over the downstream 47S region. Interestingly, our unpublished as well as published alignments of public ChIP data sets also identify the SpPr region as a site of H3K4me3 and CTCF binding [59], suggesting that this region defines a boundary between upstream repressive chromatin and the Enhancer/47S region. We cannot directly address the state of the methylated genes, but published data suggest they lie in the inheritably silenced NORs and are in a classical heterochromatin state [20], [58]. Our data then suggest that UBF is in greater part responsible for the dynamic transition between a potentially active and an actively transcribed rRNA gene state.

Ribosome biogenesis depends directly on the rate of rRNA synthesis, which is itself subject to stringent growth and nutrient dependent controls in both microorganisms and higher eukaryotes [60]–[62]. Levels of the many hundreds of components required for ribosome assembly are coordinated with the rate of rRNA synthesis. In bacteria, 5S rRNA is generally co-expressed with the other rRNAs, while excess ribosomal protein inhibits further translation of the corresponding mRNAs [46], [47]. If such feedback controls exist in mammals, shut down of rRNA synthesis by UBF elimination would be expected to suppress the expression of the other ribosome components. In contrast, we observed no significant change in the levels of the mRNAs required for ribosome biogenesis, suggesting that, as in microorganisms, regulation is exclusively translational or posttranslational. We also failed to observe any inhibition of *de novo* 5S rRNA, tRNAs, U3 processome RNA, and U1 and U2 splicing RNA synthesis. Conditional inactivation of Rrn3 in yeast also had little effect on 5S synthesis despite it shutting-down major rRNA transcription [63]. Thus, the level of pre-rRNA synthesis in mouse clearly does not feedback on gene expression by either RPII or RPIII, at least for the genes required for ribosome biogenesis. This suggests that coordination of ribosome biogenesis with growth and nutrient availability is achieved in a “top-down” manner via signaling networks, e.g. see [64] and/or by degradation of excess product, as appears to be the case in yeast, e.g. see [46].

Elimination of UBF caused key nucleolar proteins to coalesce into a dense nuclear body. In contrast, the rDNA dispersed into nuclear foci similar in number to the expected number of chromosomal rDNA loci and similar in size to metaphase NORs. Since it is generally believed that nucleolar components disperse during cellular stresses that shut-down rRNA synthesis [2], , the formation of a well-defined nucleolar body independent of the rDNA was quite unexpected. The formation of these nucleolar bodies reveals the existence of storage sites for nucleolar components within the mammalian cell nucleus. They are clearly distinct from the pseudo-NORs since these form around large synthetic arrays of rDNA repeats [66], while the nucleolar bodies do not depend on an underlying rDNA locus. However, our data strongly support the view that UBF is indeed the key factor in recruiting nucleolar proteins to the rDNA [67]. The nucleolar bodies are probably related to the nucleolar precursor bodies of pre-implantation embryos, which also exist independently of the rDNA loci, and may be related to subnuclear structures such as the Cajal, Coiled, PML and Nuclear bodies [68], several of which have been implicated in aspects of nucleolar function. They could also play important roles during normal cell division, when rRNA synthesis is shutdown and active nucleoli are no longer evident.

## Materials and Methods

### Generation of UBF conditional mutations

Fragments from the *Ubf* gene (Ensembl ENSMUSG00000020923 (GRCm38:11:102303960:102320342:-1)) covering exon 2 (coordinates 102307100–102308992), exons 3, 4 and 5 (102308989–102310581), and exons 6 and 7 (102310611–102313115) were isolated from isogenic ES cells (WW6 [69]) and inserted into the recombination vector pGKneoF2L2DTA [70] as indicated in Figure S1A. The resulting construct (pGK3'Del5'c2) was linearized and used to electroporate WW6 ES cells, which were then selected with G418. Resistant clones were amplified and analyzed by Southern blotting to identify correctly recombined clones. These clones were then used to generate two independent mouse lines using the services of the McGill and CRCUL Transgenic Core Facilities. Subsequent crossing to induce recombination of FRT and Lox sites and introduction of an ER-Cre used the following mouse strains from Jackson Laboratory; FLPeR (#003946), Sox2-Cre (#004783) and ER-Cre (#004847).

### Embryo collection, culture and genotyping

Heterozygous *Ubf* ^Δ/*wt*^ mice were inter-crossed and embryos isolated from pregnant female at 3.5 dpc by flushing uterine horns with *DMEM*. For *in vitro* development, embryos were incubated in ES cell media ((DMEM, 10% fetal bovine serum, 1% L-Glutamine, 1% Penicillin/Streptomycin, 0.1 mM β-MeOH) and cultured for one day in 5% CO_2_ at 37°C. Embryos were collected in 8-wells plate (*Ibidi*) and imaged by bright-field microscopy. DNA from the individual blastocysts was then amplified using the *REPLI-g Mini kit* (QIAGEN) and individual embryos were genotyped by PCR using the primers: A; 5′TGATCCCTCCCTTTCTGATG, E; 5′ATCTAACCCCGCTTTCCTGT, C; 5′CACGGGAAAACAAGGTCACT, see Figure 1A and S1A.

### Isolation of UBF-conditional MEFs

Primary mouse embryo fibroblasts (MEFs) from E14.5 *Ubf^fl/fl^/Er-cre^+/+^* and isogenetic *Ubf^wt/wt^Er-cre^+/+^* embryos were prepared as previously described [71]. They were cultured in Dulbecco's modified Eagle medium (DMEM)-high glucose (Invitrogen), supplemented with 10% fetal calf serum (Wisent), L-glutamine (Invitrogen) and Antibiotic/Antimycotic (Wisent). MEFs were immortalized by the introduction of the SV40 Tt antigens via transfection using the pBSV0.3T/t, a modification of the pBSV-early vector [72] kindly provided by E. W. Khandjian.

### Inactivation of *Ubf* in cell culture, and analysis of genotype, RNA and proteins

Cells were grown in 6 cm petri dishes (0.8×10^6^ cells each) for 18 hours in DMEM, high glucose, 10% fetal bovine serum. To activate ER-Cre, 4-hydroxytamoxifen (4-HT) was added to a final concentration of 50 nM, and after 4 hr incubation the medium replaced with fresh medium without 4-HT and cells harvested for analysis at various time points. This minimal 4 h ER-Cre induction generates full recombination of Lox sites while avoiding the non-specific effects of more common treatments with 0.6–2 µM 4-HT for 24–48 h [73]. Analyses of RNA, protein and genotype were systematically carried out on parallel cell cultures. Cells were genotyped by PCR before and after 4-HT treatment using the primers: A; 5′TGATCCCTCCCTTTCTGATG, B; 5′TGGGGATAGGCCTTAGAGAGA, C; 5′CACGGGAAAACAAGGTCACT, (Figure 1A). Metabolic labelling of RNA was carried out just before cell harvesting by addition of 10 µCi [^3^H]-uridine (PerkinElmer) to the culture medium and incubation for a further 3 h. RNA was extracted with Trizol (Invitrogen) according to the manufacturer's protocol and analyzed by gel electrophoresis, fluoroimaging (ENHance, PerkinElmer) and RNA species quantitated by scintillation counting as previously described [25], [26]. For total protein, cells were washed with cold PBS, scraped into PBS, centrifuged 30 s at 14 000 r.p.m., then resuspended in sodium dodecyl sulphate (SDS) loading buffer. After fractionation on 8%, 12% or 5–15% gradient SDS–polyacrylamide gel electrophoresis (SDS-PAGE [74]), cell extracts were analysed by standard Western blotting procedures.

### Antibodies for western, immunofluorescence and ChIP analyses

Rabbit antibodies against UBF, RNA Polymerase I (RPI) large subunit (A194) and TTF1 were generated in the laboratory, anti-Rrn3/TIFIA, -TAF1C and –TAF1B were kindly provided by Ingrid Grummt and anti-H1.4 and -H1.4-S187phospho kindly provided by Craig Mizzen. All other antibodies were obtained commercially; Anti-TBP, -H2A.Z, -H3 and -H4 (Abcam), anti-H1, -H3K9met3, -H4ac5 and -HP1α (Millipore), anti-Tubulin (Sigma), and anti-Fibrillarin (Covance). The pre-immune (PI) serum was from the rabbit in which the UBF antibody was generated.

### Chromatin Immunoprecipitations (ChIP)

ChIP was performed essentially as previously described [75]. Cells were fixed with 1% formaldehyde for 10 min at room temperature. Nuclei were isolated using Lysis Buffer (10 mM Tris pH 7.5, 10 mM NaCl, 3 mM MgCl2, 0.5% NP-40), the resulting chromatin sonicated (Bioruptor, Diagenode) for 25 min in IP Buffer (150 mM NaCl, 50 mM Tris-HCl pH 7.5, 5 mM EDTA, 0.5% NP-40, 1% Triton X-100), and used immediately without freezing. Each immunoprecipitation was carried out on the equivalent of 16×10^6^ cells. Immunoprecipitated DNA was analysed by qPCR/SYBR Green. Reactions (20 µl) were performed in triplicate with 2.5 µl of sample DNA, 20 pmol of each primer, and 10 µl of Quantitect SYBR Green PCR Master Mix (QIAGEN). Forty-five reaction cycles of 30 s at 94°C, 30 s at 58°C, and 30 s at 72°C were carried out on a Multiplex 3005 Plus (Stratagene/Agilent). The amplicon coordinates relative to the 47S rRNA initiation site (BK000964) were as follows: IGS3, 42646–42903; SpPr, 43089–43253; Tsp, 43267–43421; T_0_/Pr, 45133–40; ETS, 3078–3221; ITS1, 6258–6432; 28S, 10215–10411; T1–3, 13412–13607; IGS2, 25552–25783. Two to five biological replicates were analysed by ChIP for each antibody; UBF, TTF, H4 and H4ac 5 times each, RPI, H3, H3K9me3 and H1 3 times each, TIFIA, TAF1C, TAF1B, TBP, H1.4, H1.4-p187, HP1a and H2Az 2 times each. Though it was not possible to perform all ChIP analyses in parallel on all chromatin preparations, all analyses included both UBF and TTF ChIPs as reference standards.

Data was analysed using the MxPro software (Agilent). The relative occupancy of each factor at each amplicon is given as % immunoprecipitation of the DNA input prior to ChIP. It was determined by comparison with a standard curve of amplification efficiency for each amplicon using a range of input DNA amounts and generated in parallel with each Q-PCR run. All primer pairs gave the similar amplification efficiencies (90–105%) as determined from the gradient of the curve fit. The curve fit correlation coefficient R^2^ was systematically between 0.99 and 1.0, demonstrating a near perfect fit.

### Analysis of rDNA methylation

The methylation assay was developed by Anne Rascle and Joachim Griesenbeck and kindly made available to us before publication. Briefly, the assay is based on the fact that rDNA is in greater part fully methylated or unmethylated. 20 µg genomic DNA was digested with BamHI and subsequently with either SmaI or XmaI, and then analyzed by Southern blotting using the PflMI/BamHI rDNA fragment (Figure S4) labelled by random priming.

#### Psoralen crosslinking analysis

Psoralen crosslinking was performed and analyzed as described previously [37], [38].

### Expression microarray analysis

RNA for expression microarray analysis was extracted by the acid phenol/guanidinium thiocyanate method [76], and subsequently purified using the RNeasy Plus Mini kit (QIAGEN). Expression analysis was carried out using Affymetrix Mouse Gene 2.0 ST microarrays by the Genome Québec Innovation Centre (Montréal). Data analysis was performed using the Affymetrix Power tools version 1.14.4 and R (www.r-project.org) version 2.14.0. Firstly, data were normalized using RMA-sketch. *Ubf^wt/wt^/Er-cre^+/+^* normalized gene expression was then subtracted from the corresponding *Ubf^fl/fl^/Er-cre^+/+^* timepoint before all the expressions were placed relative to time T0. We required a gene to be detected in at least one time point using a cutoff of 0.001 on the detection p-values obtained from the DABG algorithm (Affymetrix Power tools) to be used in the analysis.

### 3D immunofluorescence and FISH

Cells were washed with PBS, fixed in 4% PFA for 15 minutes and permeabilized with 0.5% PBS/Triton for 5 minutes. Incubation with primary antibody was performed for 1 h in PBS-5% BSA and cells were stained with AlexaFluor 488/568 conjugated anti-rabbit or -mouse secondary antibodies (Molecular Probes) and counterstained with DAPI. After mounting in 50% glycerol/50% 0.2 M Na-glycine, 0.3 M NaCl, 3D epifluorescent 3D image stacks were acquired using a Leica DMI6000B microscope equipped with an Orca C4742-80-12AG camera (Hamamatsu) and Volocity (Perkin-Elmer Improvision) and were subsequently deconvoluted (Iterative Restoration, Volocity). In a few cases 3D stacks were also obtained using a Leica SP5 II scanning confocal microscope. Nucleolar statistics were obtained from three independent immunofluorescence experiments in which ∼20 nuclei were analysed by a protocol established using the Volocity software. DAPI staining was used to define the nuclear volume and Fibrillarin staining to define the nucleoli and their individual volumes. 3D Immuno-FISH was performed as previously described [77] using a Cy3 labeled fragment from the mouse rDNA, (positions 20138 to 23651 in Genbank Accession BK000964.3). Colocalization of FISH and protein signals were estimated using Volocity and given by the Pearson Global Correlation [78].

### Ethics statement

All animal care and animal experiments were conducted in accordance with the guidelines provided by the Canadian Council for Animal Protection, under the surveillance and authority of the institutional animal protection committees of Laval University and the CHU de Québec. The specific studies described were performed under protocol #2011-054 examined and accepted by the “Comité de protection des animaux du CHU de Québec”. This ensured that all aspects of the work were carried out following strict guidelines to ensure careful, consistent and ethical handling of mice.

## Supporting Information

Figure S1 The mouse *(m)Ubf* gene is essential for mouse development beyond morula. A) Targeting vector and strategy for target insertion into the wild type *Ubf* gene. B) Structure of the targeted mutant *Ubf* gene, and the conditional *Ubf^−fl^* (Floxed) and deleted *Ubf* ^−Δ^ (Δ) alleles. C) Example of Southern analysis of targeted and deleted *Ubf* alleles using the 3′ probe indicated in B). D) Examples of PCR genotyping of mice tails. E) Examples of mouse embryos and genotyping at the equivalent of 3.5 dpc. *Ubf* null embryos arrest at the morula stage. F) In vitro development of 3.5 dpc embryos to late blastocysts (equivalent to 4.5 dpc) and subsequent gentotyping. In A) and B) the lettering A to E refer to PCR primers used in genotyping, and in D) to F) bracketed letters indicate the combinations of these primers used to genotype.
(EPS)

Figure S2 A) Time course of ER-Cre induced recombination at the UBF locus in *Ubf^fl/fl^/Er-cre^+/+^*cells treated with 50 nM 4-HT for 4 h. B) and C) Whole protein extracts prepared from *Ubf^fl/fl^/Er-cre^+/+^*cells at the given times post 4-HT treatment were analyzed by Western blot to determine the levels of UBF and of 13 other relevant proteins. Tracks were loaded with equal amounts of total protein with the exception of the first three tracks in B in which the given percentages of whole extract at 0 h post 4-HT treatment were loaded.
(EPS)

Figure S3 A) Map of the rDNA repeat showing the sites (amplicons) sampled by ChIP analysis. B) ChIP analysis of the individual components of SL1 used to generate the mean distribution of this initiation factor shown in Figure 3B. The data are shown as percent recovery in ChIP/Q-PCR assays of chromatin from *Ubf^fl/fl^/Er-cre^+/+^* cells prepared at the indicated times post 4-HT treatment, as in Figure 3B.
(EPS)

Figure S4 A) DNA CpG methylation assays of *Ubf^fl/fl^/Er-cre^+/+^* and *Ubf^+/+^/Er-cre^+/+^* cells at the given times after 4-HT treatment. The upper diagram indicates the BamHI rDNA fragment that was analyzed by Southern blot and the position of the probe used. The lower panels show the uncleaved BamHI rDNA fragment (4.7 kb) and the 0.9 kb fragment generated by cleavage of all 11 internal SmaI or XmaI sites. SmaI is sensitive to CpG methylation while XmaI is not. Hence, on genes on which all SmaI/XmaI sites are methylated SmaI cleavage is prevented, leaving the 4.7 kb BamHI fragment intact, while XmaI cleavage continues to generate only the fully cleaved 0.9 kb fragment. Since the 4.7 kb fragment can be cleaved at all or any of 11 SmaI sites, even fractional changes in methylation status will be detected by this technique. However, only the 4.7 and 0.9 kb fragments were detected at all time points, showing that little if any change in methylation status occurred. B) Raw preimmune subtracted ChIP data for H3, H3k9me3, H4 and H4ac. C) Raw, unsubtracted ChIP data for HP1α and H1.4-S187p and preimmune antibodies. D) Analysis of H4ac/H4 and H3K9me3/H3 ratios at the Camk2b locus normalized to 0 h pHT.
(EPS)

Figure S5 Unbiased gene enrichment plots of expression microarray data corresponding to the Boxplots shown in Figure 5A. The data indicate the relative gene expression levels for *Ubf^fl/fl^/Er-cre^+/+^* cells at 72 h post 4-HT normalized to relative levels for *Ubf^wt/wt^/Er-cre^+/+^* cells at the same time point. The original data can be found in the GEO databank under the accession number GSE55450.
(EPS)

Figure S6 A) to D) Single optical sections from 3D IF image stacks of *Ubf^fl/fl^/Er-cre^+/+^* cells stained for UBF, RPI (large subunit, A194), Rrn3/TIF1A or TTF1 and for Fibrillarin, and counter-stained with DAPI, at the indicated times post 4-HT treatment. Images show 0.2 µm optical sections from deconvoluted image stacks.
(EPS)

Figure S7 A) *Ubf^fl/fl^/Er-cre^+/+^* cell metaphase spreads were stained for rDNA by FISH (green) and counterstained with DAPI (red). B) to D) 3D Immuno-FISH of *Ubf^fl/fl^/Er-cre^+/+^* cells at the indicated times post 4-HT treatment, analyzed by FISH for rDNA (green) and by IF for UBF, Fibrillarin and TTF1 respectively, and counter stained with DAPI. Images show single 0.4 µm optical sections from deconvoluted 3D image stacks.
(EPS)
